# City Size and Permanent Settlement Intention: Evidence from Rural-Urban Migrants in China

**DOI:** 10.3390/ijerph19020676

**Published:** 2022-01-07

**Authors:** Yanjiao Song, Nina Zhu, Feng Luo

**Affiliations:** 1 The Center for Modern Chinese City Studies, Institute of Urban Development, East China Normal University, Shanghai 200062, China; yjsong@iud.ecnu.edu.cn; 2 China Institute, Fudan University, Shanghai 200433, China

**Keywords:** city size, permanent settlement intention, rural-urban migrants, agglomeration economies, crowing effect

## Abstract

The location choice and livelihoods of rural-urban migrants are critical to the sustainable development of cities. By using data from the China Migrants Dynamic Survey (CMDS) in 2017, this paper extant the Rosen–Roback’s model by adding factors of urban social network and air pollution to the function of the individual utility of migrants. Both the Probit Model and IV estimates imply evidence of an inverse U-shaped pattern of city size and migrants’ permanent settlement in urban China. This view proves that Chinese migrants like to settle permanently in large cities, but not mega-cities, such as Beijing and Shanghai. The internal mechanism is explained by the agglomeration economies and the crowing effect brought by city size. In mega-cities, the attractiveness of the city caused by wage premium cannot offset the combined repulsive force caused by the high housing price, bad urban social network, air pollution, and health deterioration. It is worth noting that air pollution has a significant negative impact on the settlement intention of migrants, such as health conditions and precipitation. Besides, there is heterogeneity among high-skilled migrants and low-skilled migrants in different city sizes. Our findings enhance the understanding of “Escape from megacities” in China and have implications for the reform of the housing security system and the exploration of the urbanization path.

## 1. Introduction

The issues related to the location choice and livelihoods of rural-urban migrants are critical to the sustainable development of cities [1,2,3,4]. In China, the influx of large-scale rural labor force into cities has promoted the optimal allocation of labor factors across regions [5]. However, the settlement of migrants is unstable and reciprocating, migrants often move back and forth in the working place and the out-flow area because of the high living cost and the deficiency of the urban public service provide for them [6]. According to the data of the Seventh National Population Census, China’s urbanization rate rises from 17.92% in 1978 to 63.89% in 2020, and the number of migrants has reached 376 million. Although China’s registered household system has been continuously liberalized in recent years, the access threshold in megacities is still very strict. Recent reforms in urban housing provision seem to overlook the needs of the migrants. Therefore, it will be interesting to observe the subjective willingness of migrants to settle permanently in the working city, to provide a perspective for realizing the “citizenship” of the floating population in developing counties.

There is abundant research on the motivation of migration, and the influencing factors can be summarized as demographic characteristics (age, gender, marital status, and education), economic factors, family characteristics, city-level factors, climate, and so on [1,3,5,7]. Scholars reach a consensus that the distribution of migrants primarily focused on the areas where jobs are available [8]. And there is a negative correlation between housing prices and migration. Helpman [9] first studied the impact of house prices on migration, and Potepan [10], Jeanty [11], and Saiz [12] verified that house prices are not conducive to migration intention. But there is a positive correlation between access to formal housing and stronger settlement intention [13]. However, as the crucial element of urban settlement associated with hukou, housing remains difficult to attain for Chinese migrants, making them hard to settle permanently in large cities [14,15]. Besides, some studies analyzed the effect of migrants’ health and their social integration in the city. Results show that physical and mental health are both important to the settlement intention of migrants, and the interaction with local urban residents is crucial for their urban integration [5,16,17]. Some scholars also studied the institutional factors and found that *hukou* had a significant negative binding force on migration due to the strict *hukou* rules in megacities [18,19,20].

Recently, the emerging body of studies begin to bring urban public welfare in understanding the location choice of migrants [4,15]. Tiebout (1956) first put forward the view that people prefer to live in communities with higher public service levels and proposed the rule of “voting with their feet” [21]. Under this role, the higher the level of urban public services, the stronger the willingness of the floating population to settle permanently [22]. More importantly, the public service level is closely related to the city size. The bigger the city size, the more resource allocation, and institutional preferences the city enjoys [2,23]. In China, large cities seem to be more attractive than small cities. The economies of scale effect promote the employment probability, and the urban wage premium further leads to the inflow of labor force choosing big cities [24,25,26]. Furthermore, considering the heterogeneity of migrants, the urban agglomeration effect has a stronger impact on highly skilled migrants [27]. However, other scholars believe that the interaction between the agglomeration effect and crowding effect leads to an inverted U-shaped relationship between city size and labor productivity [28,29]. The impact of housing cost on migration is also related to city size. In China, due to the high burden of housing prices, the crowding effect is greater than the agglomeration economics in mega-cities, resulting in a decline in the attraction of cities to migrants, especially the highly skilled migrants [30,31]. So far, there’s only one study that closely focuses on city size and migrants’ settlement intention [32]. But this research defines city size as a city as a category and analyzes the intention of hukou transfer, which is different from the migrants’ permanent settlement intention.

Based on the classical economic theory, the premise of labor migration is that the return of scale is constant [33,34,35]. The limitation of the classical economic theory is that it cannot explain the agglomeration effect brought by large cities to migrants. The “Core-peripheral Model” established by the new economic geography theory represented by Paul Krugman breaks through the constant returns to scale in classical economic theory and adds the spatial agglomeration to provide a more reasonable way for explaining the flow of labor to big cities [36,37]. Based on the previous studies, this paper makes conceptual and empirical contributions to the understanding of migrants’ urbanization in different cities. The contributions of this paper are as follows. First, this paper expanded the Rosen–Roback model, and include the social relationship network and air pollution closely related to the city size into the model, which is more in line with the welfare needs of migrants; second, in addition to the spillover effect of wages, this paper finds that city size also brings the spillover effect of social capital to the migrants, that is, the interaction term between the social network of local people and city size is significantly positive, which reveals that the promotion effect of local social network on settlement intention will be significantly strengthened with the city size. Third, in the analysis of housing cost, this paper also distinguishes the impact of rent and housing price on settlement intention. Our results reveal that Chinese migrants are very concerned about the acquisition of housing ownership. The permanent settlement of China’s floating population, both rental cost and house prices will have a significant inhibitory effect.

The remainder of the paper is structured as follows. Section 2 introduces the theoretical analysis framework. Section 3 reports the data source, empirical model, and variables statistics. Section 4 reports the empirical results, the endogenous and robustness checks. Finally, Section 5 draws the main conclusions.

## 2. Theoretical Analysis Framework

It is widely documented that large cities seem to be more attractive than small ones [3,20,38]. Why do people prefer to live in one city instead of another? The choice depends on which city’s amenity values will maximize the individual utility [39]. Based on Rosen and Roback’s theory, an analytical framework of general spatial equilibrium, which is supplemented by Glaeser, is proposed [40,41,42]. The model assumes that there is an equilibrium between labor wages, living costs, and urban amenity when the basic assumption such as sufficient labor migration is satisfied. The living cost is closely related to the housing price and the rent cost, and the bad urban crowded environment [43]. In recent years, the factors of climate amenity related to quality-of-life have been added to urban settlement decision-making [44]. Albouy D. [45] proposed adjusted amenity-value estimates which indicated that climate amenity such as mild seasons, sunshine, and coastal proximity account for most inter-metropolitan quality-of-life differences in the U.S. After that, Albouy et al. [46] present a hedonic framework to estimate US households’ preferences over local climates. Meanwhile, Imbert [47] provides new evidence on the costs and benefits of rural-urban migration in developing countries. In addition to higher living costs, the non-monetary costs of harsh living and working conditions in the city are the main barrier to migration.

Based on the analysis above, we extant the Rosen–Roback’s model for the equilibrium between wages, living cost, social network, and natural amenity to the concept of location choice of migrants in China. The model assumes that the labor mobility is free, the one who intends to permanently settle in the host city is decided by the utility function which includes the wages, living cost, social network, and natural amenity. The utility function and the constraints condition equation are then defined as:

(1) $$maxU\left(W_{t}^{i},H_{t}^{i},S_{t}^{i},N_{t}^{i}\right)={\left(W_{t}^{i}\right)}^{1-\alpha }{\left(H_{t}^{i}\right)}^{\alpha }S^{i}N^{i},\left\{C_{t}^{i},H_{t}^{i}\right\}\;$$

(2) $$s.t.C_{t}^{i}+P_{t}^{i}H_{t}^{i}\leq W_{t}^{i}$$

where *U* represents the utility of the rural-urban migrants working in the city, $W_{t}^{i}$ is the labor productivity in the city *i*. $H_{t}^{i}$ is the housing cost in the city *i* in the period of t, while we treat the price of other commodities standardized to 1. The number of general commodities and houses consumed by each migrant in the *t*-period are respectively by $P_{t}^{i}$ and $C_{t}^{i}$. $S_{t}^{i}$ represents the non-monetary factors, such as social network, and $N_{t}^{i}$ indicates the PM_2.5_ , annual temperature, and annual precipitation in the city. The agglomeration economy has a positive impact on urban wage premium. The larger the city size, the higher the wage premium. Migrants have to pay a certain proportion of their wages in exchange for living opportunities in large cities [4].

Based on the above analyses, we draw a conceptual framework for migrants’ willingness to settle permanently in the context of city size. The choice of location is the result of various factors. The factors that influence the choice of settlement intention among migrants include characteristic factors and unobserved effects. By using the classic binary logit method, this study analyzes the mechanism of the inverted U-shape trend of city size and the permanent settlement intentions of rural-urban migrants. This study uses domestic statistical data of Chinese migrants to address several questions on the heterogeneity of city size in China. What is the interpretation of each factor that leads to the inverted U-shaped trend of city size and settlement intention? How will the factors change that is related to the permanent settlement intention based on city size?

## 3. Data and Methodology

### 3.1. Data 

The individual microdata used in this paper is derived from the *China Migrants Dynamic Survey* (CMDS) published by the Migrant Population Service Center of the National Health Commission in 2017. This survey covers questions that relate to the urban public service and urban integration of the migrants who have currently resided in their immigratory city for more than one month but have not registered in the destination city. So far, the CMDS is the most detailed micro-level survey data about Chinese migrants. The samples were obtained using the Probability Proportionate to Size Sampling (PPS) method with hierarchical, multi-stage, and scale proportions used, and they were representative, which provided national data covering 31 provincial areas in China. The questionnaire includes demographic characteristic variables, family economic variables, and individual future settlement intention, in which migrants are defined as those aged 16–59 who do not have a local Hukou but have lived in the city for more than six months. The urban housing price data is from the 2016 National Information Center macroeconomic and real estate database (http://www.crei.cn, accessed on 16 June 2017) (Because the migrant questionnaire mentioned above was investigated in April 2017, and the questions on economic behaviors such as family income and expenditure in the questionnaire refer to last year, the urban housing data in this paper selects 2016 instead of 2017), and the urban population and city-level data are from the China Urban Construction Statistical Yearbook in 2017. The concept of the city is defined by the statistical data of the municipal districts in the main urban area, that is, the city includes only the urban areas, excluding the counties under the jurisdiction of the city. The index of air pollution is measured by the concentration of PM_2.5_ (in μg/m^3^), and the data mainly comes from the NASA’s Global Annual PM_2.5_ Grids data in 2017, with the spatial resolution is 0.01° × 0.01°. The CMDS questionnaire contains the city code information of individual residence. After matching with the city level data of housing price, a total of 286 prefecture-level cities are obtained in this study. Besides, to accurately verify the effect of city size on individual settlement decisions, we mainly focus on the active migration that occurs for the reason of working or doing business, accounting for 83.97% of the total sample. And for those passive migrants such as taking care of their family members, moving with their spouses, or living with relatives and friends are not included in this study. After matching, the total number of effective samples in this paper is 99,829. 

### 3.2. Empirical Framework

#### 3.2.1. Binary Probit Model

The settlement intention of migrants in this paper is not a continuous variable, but a choice variable and a dummy variable. The traditional OLS method is mainly for linear regression, and not suitable for verifying the dummy variables in this paper. Therefore, the binary *Probit* model is used for our empirical analysis in testing the effect of city size and migrants’ permanent settlement intention, which is expressed as:

(3) $$y_{i}^{*}=\alpha_{i}+\beta_{i2}CITYSIZE+\beta_{i2}X+\varepsilon_{i}\;$$

(4) $$y_{i}=\left\{\begin{array}{c} 1,y_{i}^{*}>0\\ 0,y_{i}^{*}<0 \end{array}\right.$$

where the subscript *i* refers to the investigated individual in the local city. The error term *ε_i_* denotes the unobserved factors, with a constant variance assumption. The dependent variable y in the model is measured by the migrants’ permanent settlement intention in the city, which is measured by two survey questions as to “Do you intend to stay in this for some time in the future?” and “How long do you expect to stay here?” In the original questionnaire, there are three types of answers for the urban settlement intention, which are coded as one if yes, two if no, and 3 if having no ideas. Based on the above two questions, we define the group who intends to stay in the city for at least five years as 1, while for groups who settle temporarily, do not intend to settle here, and have no idea, their permanent settlement intention is defined as 0. 

The key independent variable citysize in Equation (3) is measured by the urban resident population in the urban area. Since the urban area of the city can better reflect the agglomerative effect than the administrative area of the city, the population of prefecture-level counties and villages is not included in our study. Based on the statistical data from *China Urban Construction Statistical Yearbook,* the city size mainly refers to the sum of the registered residence population in urban areas and the temporary population in urban areas. The set of control variables X includes demographic variables, wage, and employment status, urban amenity. The demographic variables include age, gender, marital status, and educational level. Education is divided into four groups: illiterate/primary school (the base group), junior school, high school, college, and above. Considering that having a homestead in a hometown may not be conducive to migrants settling in the city, this paper controls the factor of whether there is a homestead in migrants’ hometowns. Following Shi et al. [43], we select a set of variables to measure the urban amenity, including housing cost, road density, and urban public services. For the variable of road density, the road length per capita is used to reflect the degree of road congestion in a city. Besides, this paper subdivides housing cost into housing price and rental price for comparative analysis to control the impact of housing ownership on migrants’ permanent urban settlement. In addition to controlling the effect of wage, housing cost, and road congestion, this paper innovatively adds the variable of urban social network, which is defined by whether the migrant has the closest communication with local registered residents in their spare time. In addition to the above factors, the influence of the natural environment on human behavior is also of great importance, therefore the natural amenity referring to air pollution, green land per capita of the city, temperature, and precipitation are also considered in the binary Probit model. Table 1 provides further variable definitions and descriptions.

Table 2 provides the descriptive statistics for the variables. Among the migrants, nearly half of them want to settle permanently in the city. From the overall trend of migration, the migrants are young, with an average age of 35 years old. The average duration length of migration is six years, and the average monthly wage is 4146.61. The wage level has significant group heterogeneity, with the lowest value of 0 and the highest value of 20,000 yuan. Besides, 75% of the migrants own the homestead in their hometown. For the social network in the city, gathering with fellow villagers from the same hometown in their spare time is the first choice, and such group accounts for nearly half. Only 27% of the migrants chose to have the closest contact with residents, and the remaining 20% of the migrants didn’t communicate with anyone. There is also significant heterogeneity in housing prices. The lowest urban house price is 2517 yuan per square meter, while the highest one is 45,147, with a difference of nearly 20 times. 

This paper further divides the total sample into two subsamples, that is, the group who intends to permanently settle in the city and the group who does not intend to settle. The comparative analysis of the characteristics of these two groups is shown in Table 2. The results show that there are significant differences between the two samples in terms of migration duration, wage, the proportion of home homestead, and urban social network. The longer the time of migration duration, the higher the wage level, and the closer the communication with local registered residents, the more conducive it is for migrants to settle permanently in the city. Owning a home homestead hinders the urban permanent settlement. For those who do not intend to settle permanently in the city, 80% of them still own the homestead in their hometown, which is significantly higher than that of those who intend to settle permanently in the city. 

#### 3.2.2. IV Probit Model

Considering that the willingness of the floating population to settle permanently in the city will further enhance the scale of the city, there may be a reverse causal relationship between the two variables. To overcome potential endogenous problems, we also adapt to an instrumental variable estimation (IV). IV regression is presented with the following model specifications,

(5) $$expr=\mu +\pi_{i}Z+\gamma_{i}X+\tau_{i}$$

where *X* is the control variables set as previously discussed, and *Z* is defined as a vector of the instrumental variables. The *COV(Z*,*ε_i_)* in Equation (5) equals zero to satisfy the condition that the tool variable is valid. We then replace the independent variable citysize in Equation (3) with the right side of Equation (5), with the identification of the *β_1_* coefficient as our primary interest.

## 4. Results

### 4.1. The Spatial Pattern of Migrants’ Urban Settlement Intention

**Figure 1:**
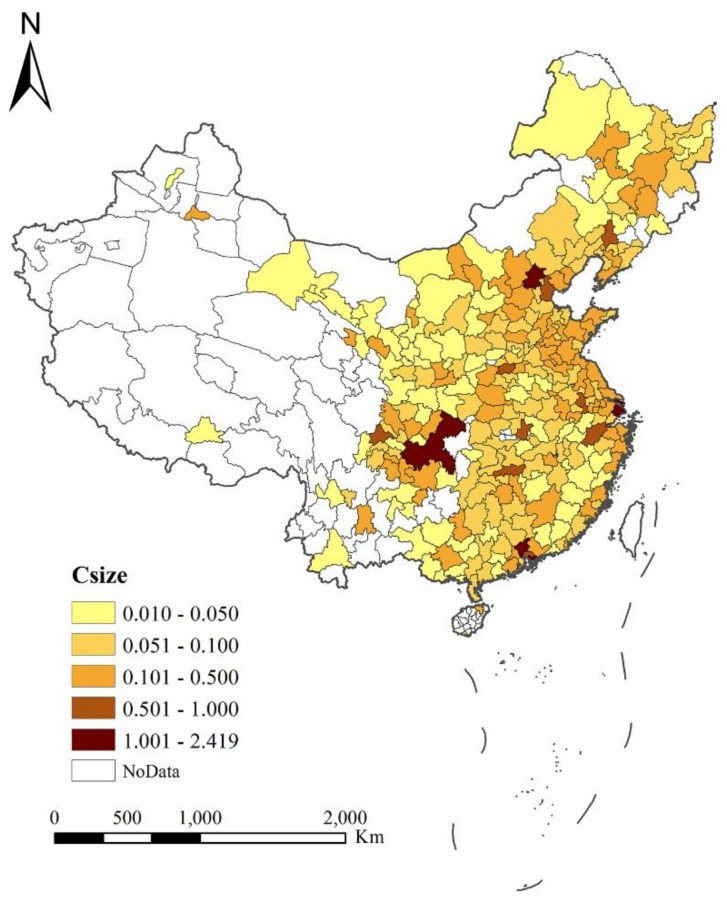
The spatial pattern of urban size in urban China.

As shown in Figure 1, the top cities with the largest city size are Shanghai, Beijing, Chongqing, and Shenzhen, etc. Overall, the cities that we use are mainly located on the right side of the Hu Line, also known as the Chinese population density distribution line. The line connects Heihe City in Heilongjiang Province in Northeast China and Tengchong County in Yunnan Province in Southwest, which is a basic straight line included 45 degrees across China. Based on the sample size weight and the probability of migrants’ urban willingness to settle in each city, this paper calculates the weight of permanent settlement intention for migrants in 286 cities (The city size is calculated in ten million. The denominator is the total number of migrants in the city, and the numerator is the number of migrants choosing to settlement permanently) and find that the top three cities with the highest proportion of migrants’ willingness to permanently settle are Jieyang in Guangdong province, Rizhao, and Liaocheng in Shandong province. What we find interesting is that in the largest cities, the proportion of migrants’ permanent settlement intention is not the highest. There is a non-linear relationship between city size and the settlement intention of migrants. Besides, from the perspective of spatial distribution, the migrants’ settlement hotspots are mainly distributed in the Yangtze River Delta, Shandong Peninsula, Pearl River Delta, Northeast China, and other major cities. 

**Figure 2:**
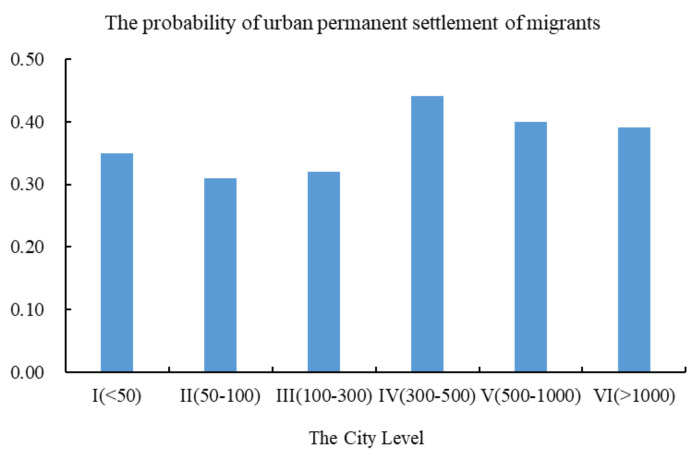
City size and the probability of settling permanently in the city.

To further show the relationship between city size and settlement intention of migrants, we divide 286 cities into six levels according to the city size and compares the probability of settlement willingness in different city levels. Figure 2 shows that the probability of permanent settlement is the highest when the city size reaches 3–5 million. 

### 4.2. The Inverted U-Shaped Relationship of City Size and Urban Settlement Intention 

Table 3 presents the estimation results on the correlation between city size and the urban settlement intention of migrants. In Model 1, only the individual demographic variables, the *hukou* status, the flow duration, and the employment are included as control variables. Model 2 controls for the city-level factors, and natural amenities. Model 3 further considers the impact of housing ownership on settlement intention and replaces the rent variable in Model 2 with housing price. Model 4 and Model 5 are estimated by using the logit model and ordinary least square (OLS) respectively for comparative analysis. The results in Table 3 show that the quadratic term of city size is significantly negative, which indicates the inverted U-shaped relationship between city size and settlement intention. It explains that migrants’ willingness to settle in cities will first be strengthened with the expansion of city size. However, it is not that the larger the city size is, the more attractive it is to migrants. When the city size reaches a certain value, which is estimated to be 19.19 million according to Model 5, the willingness to settle permanently will decline due to the excessive expansion of the city size. This confirms the conclusion drawn by other scholars, that is, migrants show a significant preference for large cities and the provincial capital, and small and medium-sized cities lack sufficient attraction to the location of the floating population [4].

For the control variables, there was a significant inverted U-shape between age and the settlement willingness of the migrants. According to the calculation, the optimal age when the migrants had the highest willingness to settle was 38.33 years old. The coefficient of gender also shows that male migrants are significantly more likely to settle permanently than women migrants. Both the coefficients of hukou status and the rural homestead on urban settlement are significantly negative. The connections of migrants in rural areas are not conducive to the integration of urban life. However, if migrants integrate into urban life and have the closest contact with the locals in their spare time, this will promote them to settle permanently in the city. The coefficient of social network with local people indicates that compared with the migrants who have the closest contact with fellow villagers, those migrants who have the most frequent contact with the locals will significantly increase their probability of settling in the city by 33%. We also find an interesting conclusion that Chinese migrants still have the traditional idea that only when they buy a house can they have a home in the city. In Model 2 and Model 3, rent has a negative effect on urban settlement. Furthermore, the housing price significantly hinders the migrants’ settlement in the city. In addition, the longer the urban roads per capita, that is, the less serious the urban road congestion, the more conducive it is for migrants to settle. For the natural amenity, the green area of the park has no obvious impact on the settlement, but migrants care about the air pollution when choosing cities to settle. The effect of PM_2.5_ on urban settlement intention is significantly negative. Compared with migrants who were not willing to permanently settle in the local cities (hereafter referred to as the “unwilling” group), the results in Model 2 indicate that precipitation had a significant negative impact on the probability of migrants being willing to settle in the city (the “willing” group), while temperature had a significant positive impact on the settlement intention. That is, cities with less rainfall and warm weather were found to be more attractive to the migrants. The empirical results of health conditions show that when migrants in illness or unhealthy conditions in the past year, the demand for urban health services will increase, which is more conducive to promoting migrants to settle permanently in cities. Besides, the coefficient of health archives is significantly positive, indicating that the establishment of health archives in cities is conductive to the urban settlement of migrants.

### 4.3. Endogeneity Bias

Due to the reverse causality between city size and settlement intention, and the problem of missing variables in the empirical model, this paper further uses the instrumental variable method to solve the endogeneity bias. The instrumental variable of city size used in this paper is the historical urban population in 2002, mainly from the earliest statistical data of the China Construction Statistical Yearbook. When matching the data, it was found that the city name and geographic administrative boundaries of some cities have undergone great changes. For example, the organizational system of Rehe province was abolished in 1955, and Chengde city began to belong to Hebei Province. The population of Chengde used in this paper is based on the data of Chengde City in the Rehe province. The Cangzhou city corresponds to the Cangxian county, while Xiangfan corresponds to Xiangyang City in the Cangxian district. After filtering and matching, a total of 274 cities are obtained. In the first stage of regression, we use the city size in 2002 and the square of city size in 2002 to replace respectively the original independent variables of city size and city size square. The first stage regression in Table 4 shows that there is a significant positive correlation between city size in 2002 and that in 2017. By using the IVregress and IVProbit estimation, respectively, the inverted U-shaped relationship between city size and urban settlement intention of migrants is still significant in Model 6 and Model 7. To test the effectiveness of the instrument variables used in this paper, the results of the weak identification test are also reported in Table 4. For the IVreg2 estimation, the Cragg-Donald F-statistic of the weak instrumental variable test is 960, which is much greater than the critical value of 7.03 under 10% bias and the assumption of weak instrumental variable is rejected. For IVProbit estimation, the result of the Wald test of exogeneity shows that the value of Chi2 (2) is 217.59. According to the rule of thumb calculated by Stock and Yogo [48,49], the F statistic of the Wald test is greater than 10, indicating that the independent variable is endogenous at the significance level of 1%, and the instrumental variable used in this study is effective.

### 4.4. The Robustness Checks and Heterogeneity Test

For the robustness test, we examined the urban area, which is expressed as the administrative boundary of the city, as an independent variable instead of the urban population. The results of Model 8 in Table 5 indicate that the relationship between the urban area and settlement intention also showing a significant inverted U-shape. In addition, for migrants, choosing to settle permanently in the city also means that they have a positive sense of urban identity. In Model 9, the dummy variable of whether migrants have a sense of citizen identity in the questionnaire is used as the dependent variable (1 = yes, 0 = no), to further test the robustness of previous results. The results show that with the expansion of city size, citizen identity tends to strengthen first and then weaken the settlement. The relationship between them also shows a significant inverted U-shape, which verifies the previous empirical results.

Skilled migrants are becoming an increasingly important element in global migration flows. in OECD countries, Skilled migrants now comprise nearly 29% of all migrants [50]. Based on the previous research, the agglomeration effect of city size is heterogeneous for different groups, and the urban agglomeration effect has a stronger impact on the high-skilled labor force than that of the low-skilled one [51,52,53]. Therefore, the heterogeneity of the migrants is indeed a crucial consideration for analyzing the settlement intention of migrants [1]. According to the question of education level in the questionnaire, high-skilled migrants as shown in Table 6 are defined as those who have finished college, or university, or received a master’s degree and above, while the others with high school and below education level are considered as low-skilled migrants. Statistics show that the high-skilled group accounts for only 10.46% of the whole sample. The Probit empirical results indicate that city size has a significant inverted U-shape effect on the settlement intention of migrants for both the high-skilled group and low-skilled group. Model 8 describes the impact of excellent air quality on the behavior of elderly migrants. The cities with PM_2.5_ values lower than 50 were defined as being in the excellent weather category, while the cities with PM_2.5_ values greater than 50 were regarded as being in the reference group. But the size and the highest position of the two U-shapes are different. For highly skilled migrants, the city size with the highest willingness to settle is 19.27 million people. The optimal city size for low-skilled migrants is smaller, which is 14.14 million people. For the control variables, housing price has a significant inhibitory effect on the settlement intention for both the high and low-skilled migrants. Moreover, contacts with local people and good air quality are also conducive to the settlement of these two groups.

### 4.5. Why Do Migrants Not Tend to Settle Permanently in Megacities?

**Figure 3:**
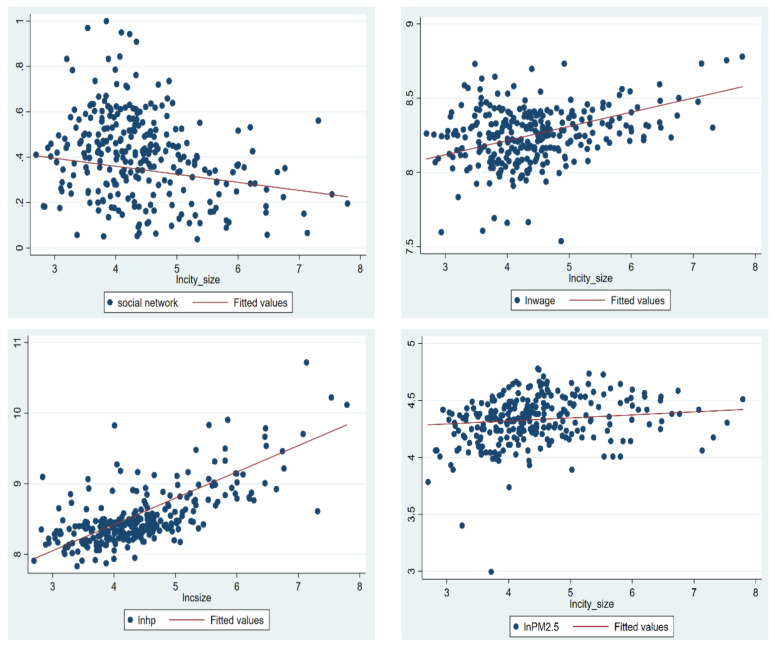

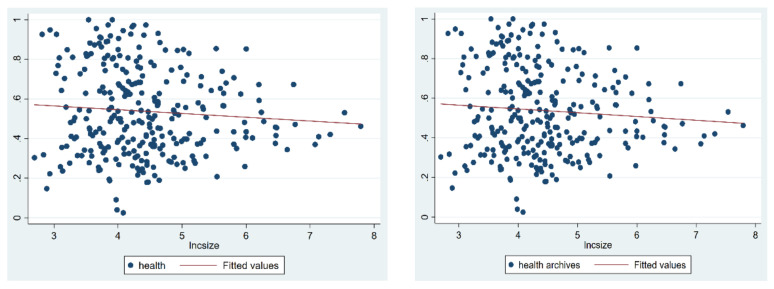
The spatial agglomeration economics and crowding effect of city size.

Besides economic conditions, comprehensive factors such as urban amenity have increasingly become important determinants of people’s settlement choices [3]. With the expansion of city size, the agglomeration effect and crowding effect brought by cities to migrants are comprehensive, which makes the comfort level of migrants in different city sizes heterogeneous. Once the city size exceeds the peak, the crowding effect is stronger than the agglomeration effect, and the probability of urban settlement intention will decrease. As shown in Figure 3, there is a wage premium for migrants. The monthly wage earned in the local city is increasing with the city size. On the other hand, the increasing size of the city also brings higher housing costs to the migrants. Previous findings suggest that the land supply index in mega-cities, such as Beijing and Shanghai, is strictly controlled, and the land supply is insufficient, resulting in housing markets being less efficient for migrants [54]. From the Figure on the left of the second line, we can see that the top three points with the highest housing prices are located at the rightmost end of the *x*-axis, and the linear slope between housing price and city size is significantly higher than that of wage and city size. In addition to the high housing cost burden, migrants also face the lack of urban social networks, air pollution, and health deterioration caused by urban expansion (See the figures on the right of the first to third lines for details).

So, to what extent will city size affect these factors and ultimately affect the settlement willingness of migrants? To explore the internal mechanism of city size on settlement intention, Table 7 introduces the interaction term between urban scale and factors such as wages, housing prices, urban social network, and air pollution. In Model 14, the marginal effect coefficient of the interaction term of city size and wage is significantly positive, indicating that the promotion of wage on permanent settlement intention increases with the expansion of city size. The marginal effect coefficient of the interaction term of city size and housing price is also significantly positive. Combined with the negative effect of housing price on settlement, the positive effect of City_size × Ln (housing_price) shows that the resistance of housing cost to permanent settlement increases with the expansion of city size. From the coefficient of interaction terms of city size and urban social network, the crowding effect caused by city size expansion also exists significantly. The combination of positive agglomeration effect and negative crowding effect brought by city size determines whether a city is suitable for migrants to live permanently.

## 5. Conclusions and Discussion

In China, the quality of amenity that a city offers matters for the permanent residential choice of migrants. Using 2017 microeconomic data from the CMDS, this study used binary Probit model analysis to explore the effect of city size on the urbanization process in China. In general, results from the descriptive statistical results show that the longer the time of migration duration, the higher the wage level, and the closer the communication with local registered residents, the more conducive it is for migrants to settle permanently in the city. After controlling for the demography characteristics, health condition, urban amenity, and natural amenity, we find that there is an obvious inverted U-shaped relationship between city size and permanent settlement intention of Chinese migrants.

The empirical results show that two mechanisms are responsible for the influence of city size on migrants’ permanent settlement intention, that is, the agglomeration effect, and the crowing effect. The importance of amenities such as wage premium and social network externalities in urban markets are linked to city size. Both the coefficients of the interaction of city size and wage and the interaction of city size and urban social network are significantly positive, showing that city size not only brings the spillover effect of human capital investment but also produces the externality of social network on permanent settlement intention of migrants. On the other hand, city size also brings a crowding effect to migrants. Housing prices have a side effect on the migrants’ willingness to permanently settle, and so is air pollution. The coefficients of the interaction of city size and housing price, the interaction of city size and air pollution, and the interaction of city size and health are significantly positive, indicating that with the expansion of city size, the crowding effect of high living cost, air pollution, and health deterioration is significant for rural-urban migrants.

The findings in this paper also have important implications for development policy. On the one hand, the natural environmental factors and individual health conditions play a key role in the settlement of migrants. The larger the city, the worse the health condition of migrants. Air pollution is also not conducive to immigrants settling in cities. In brief, our results suggest that migrants tend to settle in cities with good weather, that is, cities with low air pollution, less rainfall, and mild temperature. Besides, the urban integration between migrants and local people still needs to be improved. Our results indicate that the larger the city size, the role of the urban social network in promoting the permanent settlement of migrants is more obvious. Moreover, about 20% of migrants do not have any social activities in their spare time, which is not conducive to the mental health and the stable settlement of migrants in the city. Therefore, improving working and living conditions for migrants in urban areas may go a long way. According to the needs of the migrants, the local governments should provide more community activities or activities for promoting fellowship for migrants to help them integrate into the living city.

On the other hand, while most policymakers are concerned about improving the urban amenities, the fact that high-quality infrastructures are still insufficient for residents when considering the negative impact of road congestion on the settlement of rural-urban migrants. Many governments, especially those in mega-cities, consider that rural-urban migrants have undesirable effects on their communities of the destination city. There is still a strict access system for migrants to become new urban residents in mega-cities such as Beijing and Shanghai. In Shanghai and Beijing, education is the key factor in registered residence admission. However, by analyzing the settlement intention of high-skilled and low-skilled migrants in our paper, the urban integration of low-skilled immigrants cannot be ignored. In the future, the occupational evaluation should be more diversified in a large city, so that this index can be incorporated into the registered residence admittance system. However, it can be seen from the CMDS data that the average time of migrants living in the urban area has exceeded six years, and migrants who are willing to settle permanently have been living in the city for more than seven years. The supply of urban public services should be linked to the size of the urban population including migrants, not just based on the residents with local hukou.

## Figures and Tables

**Table 1:** The summary of variable definitions.

	Variables	Description
Dependent variable	Permanent settlement intention	Willingness to settle in the city in the next five years or more
Independent variable	City size	Resident population of the city in the urban area
Control variables	Age	15–59 years old
	Gender	1 = male; 0 = female
	Education level	Formal education (1 = illiteracy; 2 = primary, junior middle school level; 3 = high school level and above)
	Married status	Marriage status (0 = single, including divorced and widowed; 1 = married)
	Employment	Form of employment (1 = be employed;0 = self-employment)
	Wage	The wage level of last month (Dollars)
	Flow duration	The duration of moving to the city
	Rural homestead	Having a homestead in your hometown (1 = yes; 0 = no)
	Social network	The group with the most contacts in your spare time (1 = Fellow countrymen from the same registered residence; 2 = nobody; 3 = The registered residents of the local city)
	Rent	The average monthly rental cost in the city (Dollars)
	Housing price	The average housing price per square meter in the city (Dollars)
	Road congestion	Length of road per 10,000 people in the urban area
	Green garden	The Green Park area per 10,000 people in the urban area
	Air pollution	average PM_2.5_ of the city
	Health	In the last year, you have been in good health without illness, injury, or physical discomfort (1 = yes; 0 = no)
	Health archives	Has the local government of the city established a resident health record for you? (1 = yes; 0 = no)
	Temperature	Annual temperature (°C)
	Precipitation	Annual precipitation (mm)

**Table 2:** The descriptive statistics of variables.

Whole Sample						Category of Permanent Settlement			
						Yes		No	
Var	Observation	Mean	std	Min	Max	Mean	std	Mean	std
City size	99,829	486.62	588.	14.81	2418.33	519.76	618.78	489.66	591.55
Permanent settlement intention	81,435	0.45	0.50	0	1	1	0	0	0
Age	99,829	35.23	9.45	15	59	35.91	8.52	34.59	9.67
Gender	99,829	0.57	0.49	0	1	0.57	0.50	0.56	0.49
Education	99,829	2.31	0.51	1	3	0.89	0.31	0.79	0.41
Married status	98,925	0.83	0.38	0	1	2.37	0.53	2.28	0.51
Employment	91,456	0.60	0.49	0	1	0.55	0.49	0.63	0.48
Wage	91,456	653.02	442.78	0	3149.61	730.65	535.35	609.29	373.80
Flow duration	99,829	6.01	5.76	0	41	7.57	6.20	5.12	5.20
Rural homestead	99321	0.75	0.43	0	1	0.70	0.46	0.80	0.40
Social network with local people	99,829	0.27	0.45	0	1	0.37	0.49	0.22	0.43
Rent	98,248	122.61	170.27	0	7874.02	158.84	211.22	101.47	136.63
Housing price	98,248	1565.10	1179.21	396.38	7109.76	1576.68	1207.00	1558.35	1162.61
Road	98,248	15.75	6.94	4	55	15.59	7.41	15.67	6.90
Green garden	98,248	13.79	4.06	6	45	13.82	4.17	13.72	3.91
PM_2.5_	99,829	79.57	14.22	20	119	79.08	14.65	79.91	13.98
Health	99,829	0.52	0.50	0	1	0.50	0.50	0.53	0.50
Health archives	99,829	0.29	0.45	0	1	0.32	0.47	0.26	0.44
Temperature	99,829	15.40	4.87	−0.35	24.67	15.06	4.89	15.63	4.85
Precipitation	99,829	1287.41	705.41	110.46	2778.80	1215.61	680.65	1336.50	717.75

**Table 3:** The binary Probit regression of city size and migrants’ permanent settlement intention.

Variables	Model 1	Model 2	Model 3	Model 4	Model 5
	dx/dy	dx/dy	Probit	Logit	OLS
City size	0.127 ***	0.276 ***	0.373 ***	0.133 ***	0.618 ***
	(4.78)	(10.39)	(12.18)	(12.11)	(12.15)
City size square	−0.051 ***	−0.072 ***	−0.093 ***	−0.034 ***	−0.156 ***
	(−4.29)	(−6.18)	(−7.64)	(−7.80)	(−7.80)
age	0.074 ***	0.067 ***	0.067 ***	0.021 ***	0.110 ***
	(16.66)	(15.22)	(15.15)	(14.21)	(14.95)
Age square	−0.001 ***	−0.001 ***	−0.001 ***	−0.000 ***	−0.002 ***
	(−17.28)	(−16.34)	(−16.31)	(−15.48)	(−16.09)
Gender (base group: female)	0.019 *	0.031 ***	0.030 ***	0.012 ***	0.046 ***
	(1.96)	(3.22)	(3.14)	(3.48)	(2.91)
Marital status (base group: single)	0.350 ***	0.413 ***	0.412 ***	0.140 ***	0.690 ***
	(22.13)	(25.67)	(25.63)	(25.42)	(25.43)
Primary and junior (base group: illiteracy)	0.012	0.032	0.026	0.008	0.046
	(0.34)	(0.89)	(0.73)	(0.68)	(0.77)
High school and above (base group: illiteracy)	0.425 ***	0.407 ***	0.399 ***	0.142 ***	0.655 ***
	(11.61)	(11.03)	(10.82)	(11.07)	(10.69)
Rural homestead	−0.304 ***	−0.229 ***	−0.226 ***	−0.081 ***	−0.369 ***
	(−27.51)	(−21.28)	(−20.96)	(−21.11)	(−20.87)
Flow duration	0.042 ***	0.037 ***	0.038 ***	0.014 ***	0.061 ***
	(46.45)	(41.91)	(42.61)	(44.17)	(42.23)
Employment (base group: unemployment)	−0.118 ***	−0.073 ***	−0.059 ***	−0.020 ***	−0.093 ***
	(−11.94)	(−7.43)	(−6.05)	(−5.84)	(−5.81)
Wage	0.049 ***	0.059 ***	0.063 ***	0.022 ***	0.108 ***
	(9.30)	(11.57)	(12.12)	(12.38)	(12.01)
Interaction with nobody (base group: Social network with fellow-townsman)		−0.072 ***	−0.079 ***	−0.028 ***	−0.133 ***
		(−5.95)	(−6.50)	(−6.51)	(−6.64)
Social network with local people (base group: Social network with fellow-townsman)		0.330 ***	0.314 ***	0.115 ***	0.511 ***
		(30.52)	(28.29)	(29.01)	(28.20)
Rent		−0.006 ***			
		(−3.76)			
Housing price			−0.084 ***	−0.029 ***	−0.138 ***
			(−6.92)	(−6.68)	(−6.94)
Road		0.004 ***	0.004 ***	0.002 ***	0.007 ***
		(4.68)	(4.60)	(4.95)	(4.75)
Green land		−0.001	−0.000	−0.000	−0.001
		(−1.23)	(−0.44)	(−0.65)	(−0.67)
Air pollution		−0.002 ***	−0.001 ***	−0.001 ***	−0.002 ***
		(−4.43)	(−4.08)	(−4.08)	(−4.13)
Health		−0.088 ***	−0.091 ***	−0.032 ***	−0.150 ***
		(−9.50)	(−9.83)	(−9.88)	(−9.87)
Health archives		0.137 ***	0.138 ***	0.050 ***	0.227 ***
		(13.44)	(13.54)	(13.70)	(13.59)
LnPrecipitation		−0.156 ***	−0.153 ***	−0.265 ***	−0.058 ***
		(−11.30)	(−10.99)	(−11.40)	(−11.57)
Temperature		0.006 ***	0.006 ***	0.0116 ***	0.002 ***
		(3.19)	(3.27)	(3.59)	(3.81)
_cons	−2.004 ***	−2.284 ***	−1.632 ***	−0.042	−2.731 ***
	(−21.40)	(−23.08)	(−11.80)	(−0.86)	(−11.77)
N	76273	75185	75185	75185	75185

*Note: t* statistics in parentheses *** *p* < 0.01.

**Table 4:** The results of IV estimation of endogeneity.

	Model 5	Model 6	Model 7
Variables	OLS	IVreg2	IV*Probit* *2SLS*
*First-stage regressions*			
citysize_2002	-	1.986 ***	1.738 ***
		(259.87)	(117.52)
citysize_2002_square	-	0.340 ***	0.305 ***
		(42.38)	(40.72)
*Second-stage regressions*			
City size	0.618 ***	0.208 ***	0.597 ***
	(12.15)	(16.18)	(16.58)
City size square	−0.156 ***	−0.055 ***	−0.156 ***
	(−7.80)	(−11.59)	(−11.77)
Ln (wage)	0.108 ***	0.025 ***	0.069 ***
	(12.01)	(13.06)	(11.20)
Social network	−0.133 ***	−0.027 ***	−0.076 ***
	(−6.64)	(−6.03)	(−6.02)
	0.511 ***	0.111 ***	0.301 ***
	(28.20)	(27.01)	(26.28)
Ln (housing price)	−0.138 ***	−0.058 ***	−0.172 ***
	(−6.94)	(−11.50)	(−12.07)
Air pollution	−0.002 ***	−0.001 ***	−0.002 ***
	(−4.13)	(−4.50)	(−4.43)
Health	−0.150 ***	−0.030 ***	−0.083 ***
	(−9.87)	(−8.77)	(−8.69)
Health archives	0.227 ***	0.052 ***	0.143 ***
	(13.59)	(13.52)	(13.36)
Demographic variables	Yes	Yes	Yes
Family variables	Yes	Yes	Yes
Natural amenity	Yes	Yes	Yes
_cons	0.202 ***	0.176 ***	−0.954 ***
	(3.93)	(3.28)	(−6.16)
*N*	75185	69597	69597
*Adj-R^2^*	0.101	0.460	0.460
Cragg-Donald Wald F statistic	-	960	
Stock-Yogo bias critical value		10% (7.03)	
Wald test of exogeneity(chi2(2))	-		217.59
*p*-value			0.000

*Note: t* statistics in parentheses *** *p* < 0.01.

**Table 5:** The robustness checks of the urban area and citizen identity.

Variables	Model 8	Model 9
	Independent Variable: Area Square	Dependent Variable: Citizen Identity
l_living		
Urban Area	0.086 ***	
	(16.51)	
Urban Area square	−0.004 ***	
	(−12.70)	
City size		0.302 ***
		(9.07)
Citysize_square		−0.064 ***
		(−4.96)
Ln (wage)	0.062 ***	0.019 ***
	(12.07)	(3.50)
Social network	−0.078 ***	−0.001
	(−6.48)	(−0.05)
	0.320 ***	0.392 ***
	(28.83)	(31.27)
Ln (housing price)	−0.101 ***	−0.487 ***
	(−8.56)	(−37.81)
Air pollution	−0.002 ***	−0.000
	(−6.86)	(−1.16)
Health	−0.091 ***	0.211 ***
	(−9.84)	(21.52)
Health archives	0.130 ***	0.223 ***
	(12.78)	(19.71)
Demographic variables	Yes	Yes
Family variables	Yes	Yes
Natural amenity	Yes	Yes
_cons	−1.451 ***	4.035 ***
	(−10.37)	(27.47)
N	75,185	75,185

*Note: t* statistics in parentheses *** *p* < 0.01.

**Table 6:** The heterogeneity test of city size and settlement intention among high-low skilled migrants.

Variables	Model 10	Model 11
	High-Skilled Migrants	Low-Skilled Migrants
City size	0.359 ***	0.475 ***
	(4.05)	(13.99)
City size_ square	−0.106 ***	−0.123 ***
	(−3.08)	(−9.30)
Ln (wage)	0.105 ***	0.061 ***
	(6.01)	(11.39)
Social network	−0.138 ***	−0.083 ***
	(−3.38)	(−6.53)
Excellent air quality	0.279 ***	0.311 ***
	(9.48)	(25.87)
Ln (housing price)	−0.094 ***	−0.123 ***
	(−2.86)	(−9.14)
Good weather(air quality ≤ 50)	0.214 **	0.358 ***
	(2.36)	(9.89)
Health	−0.112 ***	−0.087 ***
	(−4.18)	(−8.85)
Health archives	0.140 ***	0.145 ***
	(4.91)	(13.30)
Demographic variables	Yes	Yes
Family variables	Yes	Yes
Natural amenity	Yes	Yes
_cons	−2.521 ***	−1.059 ***
	(−6.05)	(−7.34)
N	9843	65342

*Note: t* statistics in parentheses ** *p* < 0.05, *** *p* < 0.01.

**Table 7:** The mechanism of city size on urban settlement intention.

Variables	Model 12	Model 13
	dx/dy	dx/dy
City size	0.535 ***	0.499 ***
	(14.26)	(11.47)
Citysize_square	−0.125 ***	−0.100 ***
	(−4.91)	(−3.38)
City_size × Ln (wage)	0.110 ***	0.042 ***
	(14.26)	(4.63)
City_size × Social_network	0.152 ***	0.083 ***
	(9.80)	(4.16)
City_size × Ln (housing_price)	0.176 ***	0.040 *
	(10.24)	(1.94)
City_size ×Air_quality	0.012 ***	0.009 ***
	(17.63)	(11.36)
City_size ×Health	0.052 ***	0.053 ***
	(4.11)	(3.38)
City_size ×Health_archives	0.013	−0.059 ***
	(0.73)	(−2.80)
Ln (wage)	0.114 ***	0.068 ***
	(25.05)	(12.88)
Urban social network	0.427 ***	0.324 ***
	(44.96)	(28.82)
Ln (Housing_ price)	−0.067 ***	−0.105 ***
	(−6.14)	(−8.32)
Air pollution	−0.000	−0.001 **
	(−1.53)	(−2.44)
Health	−0.097 ***	−0.094 ***
	(−12.00)	(−10.11)
Health archives	0.172 ***	0.142 ***
	(19.10)	(13.57)
Demographic variables	No	Yes
Family variables	No	Yes
Natural amenity	No	Yes
_cons	−0.673 ***	−1.673 ***
	(−6.88)	(−11.82)
*N*	75185	75185

*Note: t* statistics in parentheses ^*^ *p* < 0.1, ^**^ *p* < 0.05, ^***^ *p* < 0.01.

## Data Availability

The original data of this study was obtained from the Migrant Population Service Center, National Health Commission China. We are authorized to use the data by submitting a formal application to the Migrant Population service Center in November 2019, and the data was available online at the website http://www.chinaldrk.org.cn (Accessed on 24 November 2020).
